# EBO accredited first centre in Romania

**Published:** 2018

**Authors:** Zemba Mihail

**Affiliations:** * Editor-in-Chief

The European Board of Ophthalmology (EBO), founded in London in 1992, is a permanent working group of the Ophthalmology Subspecialty Section of the European Union of Medical Specialists (UEMS).

The Board has the role of supervising the level of education in Ophthalmology in the European Union. EBO intends to obtain standards of education, and to harmonize knowledge and training across Europe. The main mean for EBO to realize that, alongside the EBO exam, CME control, and Grants is the accreditation of centers aspiring to reach the standards required.

The National Delegates of Romania to EBO, Cornel Stefan, MD, PhD, and Assoc. Prof. Marian Burcea, MD, PhD, supported the Department of Ophthalmology of “Dr. Carol Davila” Central Military Emergency University Hospital to apply for EBO accreditation.

On the 8th and 9th April, an Evaluation Committee from EBO came to Bucharest. The members of the Committee, Tina Xirou, MD, PhD, from Athens, Greece, and Prof. Andreas Wedrich, MD, PhD, from Graz, Austria, spent two days in the Clinic, following the daily activities, mainly the activities of the residents.

**Fig. 1:**
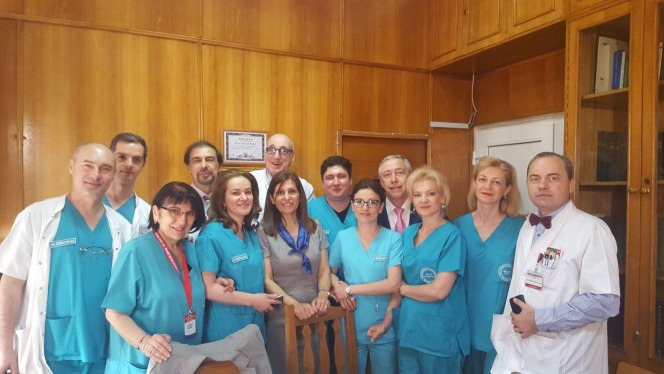
EBO assessors with the staff of the clinic

The staff involved in residents’ training was assessed (years of experience in ophthalmology, clinical activity, research activity, and experience in training). The logistics of training – dedicated spaces, access to textbooks, paper and electronic journals, internet access, time in daily schedule for study seemed to be very important for the assessors. The presence of a simulator for ophthalmic surgery with open access (for residents from the clinic, but also for residents from other centers) was much appreciated.

For almost three hours, there was a discussion with the residents. During this talk, a lot of topics were clarified: numbers of residents in clinic, number of residents in each year of training, resident/ staff ratio, number of patients/ year, number of surgeries/ year, number of patients in different areas of ophthalmology (medical retina, surgical retina, glaucoma, refractive surgery, strabismus, ophthalmic pediatrics, ophthalmic oncology, oculoplastics, cataract, medical cornea, surgical cornea), the opportunity – or not – to follow some time in each of these subspecialties.

The clinic obtained a very good evaluation for the diagnostic and therapeutic equipments, which were considered above the level of many units from western countries.

The global impression was positive and the assessors presented their data to EBO Accreditation Committee. Two months later, the Department of Ophthalmology of “Dr. Carol Davila” Central Military Emergency University Hospital became the first EBO accredited centre in Romania.

**Fig. 2:**
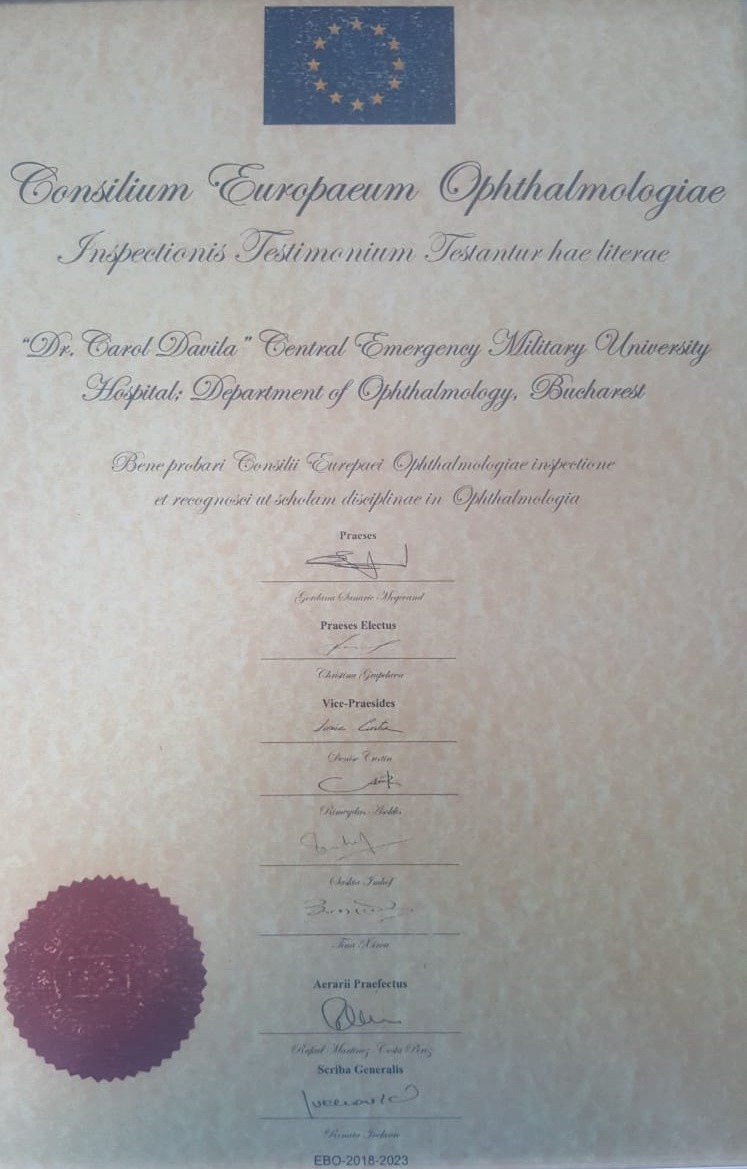
EBO Diploma

**Editor-in-Chief** **Mihail Zemba, MD, PhD**

